# The Role of Spirituality and Religiosity in Healthcare During the COVID-19 Pandemic: An Integrative Review of the Scientific Literature

**DOI:** 10.1007/s10943-022-01549-x

**Published:** 2022-03-29

**Authors:** Rocío de Diego-Cordero, Amanda Ávila-Mantilla, Juan Vega-Escaño, Giancarlo Lucchetti, Bárbara Badanta

**Affiliations:** 1 Research Group PAIDI-CTS 969 Innovation in HealthCare and Social Determinants of Health, Department of Nursing, Faculty of Nursing, Physiotherapy and Podiatry, University of Seville, 41009 Seville, Spain; 2 Department of Nursing, Faculty of Nursing, Physiotherapy and Podiatry, University of Seville, 41009 Seville, Spain; 3 Department of Nursing, Faculty of Nursing, Physiotherapy and Podiatry, University of Seville, c/Avenzoar, 6, 41009 Seville, Spain; 4 School of Medicine, Federal University of Juiz de Fora, Juiz de Fora, Brazil; 5 Research Group PAIDI-CTS 1050 Complex Care, Chronicity and Health Outcomes, Department of Nursing, Faculty of Nursing, Physiotherapy and Podiatry, University of Seville, 41009 Seville, Spain

**Keywords:** Coronavirus, COVID-19 pandemic, Faith, Health care, Religiosity, Spirituality, Well-being

## Abstract

The pandemic caused by the new coronavirus SARS-CoV-2 has been affecting populations globally since the end of 2019. Previous studies have indicated that spirituality in these times of crisis serves as a source of hope and well-being that helps people get through the day. This study investigated the role of spirituality and religiosity in healthcare during the COVID-19 pandemic. An integrative review of the scientific literature available on PubMed, Scopus, and Web of Science databases and a review of the gray literature in the Information System on Gray Literature in Europe (OpenGrey) were carried out. From a total of 1,338 articles searched; 25 articles were included in this review (22 quantitative observational, 2 qualitative and one randomized controlled trial). Our findings revealed the importance of including spirituality in clinical practice for both health professionals and patients. On the one hand, spirituality can be considered a good coping strategy used by healthcare professionals to promote mental health and well-being during the COVID-19 pandemic and resulting in greater patient satisfaction with the care given. On the other hand, addressing spiritual needs of individuals leads to a reduction in stress, anxiety, depression, and an increase in resilience and hope among patients.

**Supplementary Information:**

The online version contains supplementary material available at 10.1007/s10943-022-01549-x.

## Background

In recent decades, the field of spirituality, religiosity, and health research has increasingly consolidated in the scientific community (Lucchetti & Lucchetti, 2014), presenting an increase of 600% in the number of publications between 1993 and 2002 (Valiente-Barroso & García-García, 2010) and gaining even more attention of researchers particularly over the last 35 years (Koenig et al., 2021).

In the field of nursing, the relationship between spirituality and healthcare has been considered one of the pillars of modern nursing. According to Florence Nightingale, spirituality is considered an intrinsic component of human nature and 'the most profound resource and powerful healing power available to the person.' In fact, previous studies have shown that including spiritual care in nursing practice not only provides benefits for patients but also for nurses, since the practice of their profession brings greater satisfaction (Vlasblom et al., 2011). Despite this importance, few nurses feel prepared to handle these issues in clinical practice (Cordero et al., 2018) and there is a clear gap to address spiritual needs despite the fact that most patients want their healthcare professionals to discuss these issues (Sager, 2020).

In case of others health professionals such us physicians, they pointed out obstacles such us lack of training, lack of time, and fear in addressing this dimension in their care practice to provide adequate care in this regard (López-Tarrida et al., 2021).

Spirituality is considered a crucial resource during disaster situations. As an example, during World War I, chaplains were assigned to military units as a resource for moral support, and, during times of battle, they went to the front to give absolution to outgoing soldiers (Chirico & Nucera, 2020). Another example was the Asian highly pathogenic avian influenza HPAI A/H5N1 pandemic. In this period, a study found that spirituality was associated with higher levels of positive emotions and helping behavior and lower levels of illegal behavior (Smith et al., 2009). Likewise, in 2014, the largest Ebola epidemic in history caused 10,000 deaths and more than 26,000 people were infected in Africa. During this crisis, religiosity was also an important aspect of the provision of care (Marshall & Smith, 2015).

These aforementioned crises were followed by one of the most unprecedented infectious diseases that the world has ever faced, the COVID 19 pandemic. According to data recorded by John Hopkins University, the total number of cases of infections worldwide at the beginning of October 2021 was 237,668,106 and the total number of deaths was close to 5 million people (Center for Systems Science & Engineering at Johns Hopkins University, 2021). Likewise, the World Health Organization (WHO) reported an increase in mental health problems due to the disease itself, as well as the social distancing and confinement imposed to stop the contagion (Pan American Health Organization, 2020). In this context, spirituality and religiosity have emerged as important coping mechanisms to overcome mental and physical health problems, promoting positive emotions that could strengthen the immune system and minimize suffering (Bhaskar & Mishra, 2019).

During the COVID 19 pandemic, spiritual support has in fact become evident and due to the limited number of chaplains or due to the avoidance of contact with patients, healthcare professionals were instructed to give blessings to those who were dying from loved ones, highlighting the role of spiritual care in disaster scenarios such as the COVID-19 pandemic, to alleviate stress and psychological suffering (Chirico & Nucera, 2020).

Despite these initiatives, few studies have collected evidence on the role of spiritual and religious beliefs on care during the COVID-19 pandemic. This is particularly important to understand how spirituality is addressed and what are the challenges to address it, helping healthcare managers plan better interventions in future pandemics.

The spiritual approach in disaster situations includes holistically assessing the patient, recognizing values and beliefs, detecting spiritual needs, and offering appropriate care. The main objective of this study was to provide a comprehensive analysis of the role of spirituality and religiosity on health care for the general population during the COVID-19 pandemic.

Our review questions were as follows:

1. How was spiritual care provided by healthcare professionals during the COVID-19 pandemic?
2. How important was spirituality and religiosity to the general population during the COVID-19 pandemic?

## Methods

### Search Strategy

An integrative review of the scientific literature was carried out between January and February 2021, following the guidelines of (Whittemore & Knafl, 2005) for comprehensive reviews. These authors identified five stages in conducting an integrative review: (a) Problem identification; (b) Literature search; (c) Data evaluation; (d) Data analysis, and (e) Presentation. Before starting the study, two external reviewers separately evaluated the protocol, which was registered in Prospero (registration number CRD42021269210).

Two authors (Author 1 and Author 2) carried out the identification of the problem (Stage 1). Then, two authors (Author 2 and Author 3) independently replicated the search strategy in three electronic databases: PubMed, Scopus, and Web of Science. The search strategy used combining keywords and Boolean expression was: (covid-19 OR coronavirus OR 2019-ncov OR sars-cov-2 OR cov-19) AND (religion* OR religiosity OR spiritual OR faith) Table 1.

**Table 1:** Database

Base de Datos	Strategy	Articles found applying the strategy	Selected articles (inclusion/exclusión criteria)	Included articles
PUBMED	(*covid-19* OR *coronavirus OR 2019-ncov* OR *sars-cov-2* OR *cov-19*) AND (religion* OR *religiosity* OR *spiritual* OR *faith*)	384	73	16 valid
SCOPUS		716	44	6 valid
WOS		415	6	3 valid

Another author (Author 2) reviewed the reference lists of the selected articles, and Author 3 made a review of the gray literature in the Information System on Gray Literature in Europe (OpenGrey). Mendeley software (version 1.19.4) was used for the organization of the references in this review.

### Inclusion and Exclusion Criteria for Selected Articles

Articles were included if they: (a) investigated spiritual care provision during the COVID-19 pandemic; (b) were published in peer-reviewed journals; (c) had original data; (d) were published between 2020 and 2021 and (e) used quantitative, qualitative, or mixed designs. Only studies whose language was English or Spanish were considered. Opinion articles such as editorials or letters to the editor, clinical cases, book chapters, dissertations, essays, corrections, communications, correspondence, as well as articles without access to the full text, were excluded. The PICOTS criteria are shown in Table 2.

**Table 2:** Population, interventions/exposure, comparator, outcome, time and design (PICOTS) criteria

PICOTS criteria	
*Population*	Healthcare providers, general population and patients
Intervention/exposure	Spiritual and religious beliefs; spiritual interventions
Comparator	Those individuals with low levels of Spiritual and religious beliefs; groups with no spiritual interventions
Outcome	Improvements in physical and mental health care and well-being
Time	Published during the COVID-19 pandemic (2020 and 2021)
Study desing	Quantitative, qualitative studies and mixed methods

### Study Selection

Author 2 and Author 3 independently selected studies that met the inclusion criteria and the exclusion criteria. Initially duplicate records were eliminated, and then titles and abstracts were reviewed. Discrepancies were resolved by Author 1. The reading of the full text of the selected articles was carried out by 3 authors (Author 1, Author 2, and Author 3) in order to include studies investigating spiritual care during the Covid-19 pandemic. Author 1 was responsible for resolving any discrepancies that arose in this phase.

### Data Extraction

The extraction and analysis of the data of the articles was carried out by Author 5 and later verified by Author 4. A table of results (Table 3) was prepared independently by Author 2 and Author 3 after discussion. Finally, the data extracted were as authors, year, country, purpose of the study, design and sample, data and instruments, findings, and quality.

**Table 3:** Results

References, year, country	Purpose of the study	Research design and sample characteristics	Data collection and instruments	Major findings	Quality
Al Eid et al. (2021)	Determine the role of religiosity and hope in COVID-19 patients	Cross-sectional study (*n* = 426 COVID-19 patients)	Scales: CPRS-9, CPHS-8, CPSS-10 and CPWS-10	Religiosity and hope play a positive role in the psychological well-being of patients. Carrying out strategies based on religiosity and hope may reduce the adverse effects of the stigma associated with the virus and improve the psychological well-being of COVID-19 patients	17.8/22 STROBE
Büssing et al. (2020)	To analyze whether a group of patients with malignant tumors perceive changes in their attitudes, behaviors, and interest in spiritual matters during the COVID-19 pandemic	Cross-sectional study (*n* = 292 oncology patients)	Scales: GrAw-7, SpREUK-15, WHO-5 and MLQ	The meaning of life, trust, stable relationships, the conscious encounter with nature and moments of reflection are important themes in patients. These spiritual care approaches can easily be incorporated into more comprehensive treatment and support of tumor patients, particularly in times of pandemic restrictions	17.8/22 STROBE
Durmuş et al. (2021)	To determine the effect of the COVID-19 pandemic on fear and spiritual well-being levels of older people	Cross-sectional study (*n* = 367 individuals over 65 years old)	Scales: FACIT-Sp and C19P-S	As levels of spiritual well-being increase in older people, their somatic and psychological fears of the coronavirus decrease	17.8/22 STROBE
Fatima et al. (2020)	To assess religious beliefs and coping during the COVID-19 pandemic	Cross-sectional study (*n* = 647, 360 from Nigeria, 287 from India)	RCOPE Scale	Significant percentages of people after the COVID-19 pandemic took religious coping steps to overcome their problems. During this pandemic, positive religious coping among this communities has a high prevalence	19.3/22 STROBE
Hamilton et al. (2021)	To explore how a group of African American women who have survived breast cancer use spirituality to manage stressors during the COVID-19 pandemic	Qualitative study (*n* = 18 women)	Semi-structured interview lasting 15–45 min via phone and video conferencing platform	Spirituality enabled African American breast cancer survivors to better manage their psychological distress through increased engagement in religious activities; reliance on God for protection when fearful, feeling isolated, and in need of assistance to pay household bills; finding joy and courage from listening to gospel music and reading scripture; and finding meaning through spirituality	18/21 SRQR
Kim et al. (2021)	To examine the impact of various factors affecting nurses' mental health during the COVID-19 pandemic	Cross-sectional study (*n* = 320 nurses)	Scales: PSS, GAD-7, Family APGAR, CD-RISC-10 and spiritual support	High resilience, spirituality, and high levels of family functioning are good coping mechanisms for nurses against stress, anxiety and depression caused by the pandemic. Strengthening these coping mechanisms may improve psychological well-being during the pandemic and reduce long-term negative consequences. Nurses in good mental health will be able to provide safe high-quality patient care	18.5/22 STROBE
Kostovich et al. (2021)	To evaluate a stress reduction strategy, an-Internet-based Mantram Repetition Program (MRP), for nurses caring for hospitalized Veterans	Cross-sectional study (*n* = 15 nurses and their patients *n* = 22)	Scales: ProQOL, PONS, PONS-RN, MAAS, SWB, CSQ-8 and SPNCS	Patients described high levels of presence and great satisfaction with the care provided. After the MRP, the nurses perceived greater mindfulness, spiritual well-being and presence. Participating in a MRP could lessen stress and *burnout* and facilitate nursing presence	21/22 STROBE
Kowalczyk et al. (2020)	To examine whether the exposure to COVID-19 enhances the faith and verify the power of spirituality in the face of the coronavirus pandemic	Cross-sectional study (*n* = 324 Polish)	Online survey with self-made questionnaires	People experiencing fear, suffering or illness often experience a “spiritual renewal”, because Faith allows people to keep hope as well as feel sense of security	15.3/22 STROBE
Lucchetti et al. (2020)	To investigate the association between R/S and the mental health consequences of social isolation during the COVID-19 pandemic in Brazil	Cross-sectional study (*n* = 485 Brazilians)	Online survey with self-made questionnaires	Religiosity and spirituality (R/S) seem to have an important role on the relief of suffering, having an influence on health outcomes and minimizing the consequences of social isolation. These results highlight the importance of public health measures that ensure the continuity of R/S activities during the pandemic and the training of healthcare professionals to address these issues	19.7/22 STROBE
Mahamid et al. (2021)	To investigate the relationship between positive religious coping, perceived stress, and depressive symptoms in response to the emergence of coronavirus (COVID-19)	Cross-sectional study (*n* = 400 Palestinian adults)	Scales: PSS, PMIR and CES-D-10	Positive religious coping is significantly related to a decrease in depressive symptoms among the sample of Palestinian adults, as well as a decrease in the perceived stress of the participants. Knowing that positive religious strategies can help improve the resilience and well-being of populations affected by the pandemic, it is necessary to carry out interventions that consider the religious and spiritual aspects of people	17.5/22 STROBE
Mahmood et al. (2021)	To test the relationship between religious coping and health anxiety	Cross-sectional study (*n* = 408 Pakistani Muslims)	SHAI Scale and Religiosity Scale	Muslims in Pakistan who suffer from pandemic-induced anxiety use religiosity as a coping strategy to deal with life circumstances	19/22 STROBE
Malik et al. (2020)	To evaluate the impact of the preventive measures undertaken through Yoga practice	Cross-sectional study (*n* = 126 people who perform yoga daily for 30 days)	Telephonic interview with self-made questionnaires	Performing Yoga regularly has improved control of the mind and body and enhances well-being. It also helped in boosting the immune system which can act as a preventive measure to COVID-19	14.5/22 STROBE
Nodoushan et al. (2020)	To evaluate the physical health with the spiritual and mental health of pregnant women during the COVID-19 pandemic	Cross-sectional study (*n* = 560 pregnant women)	Scales: DASS-21 and self-efficacy	The spiritual health of pregnant women decreases when they have high stress levels. High stress and low mental health can increase factors that influence preterm and unhealthy labor	16.3/22 STROBE
Nooripour et al. (2021)	To detect the relationship between resiliency and hope with the stress of COVID-19 by mediating the role of spiritual well-being	Cross-sectional study (*n* = 755 Iranian people)	Validated scales about resiliency, hope, spiritual well-being, and stress due to COVID-19	The findings showed that spiritual well-being itself cannot predict stress of Covid-19 alone. However, this variable, along with hope and resiliency, can be a good predictor of stress	18.2/22 STROBE
Pirutinsky et al. (2021)	To explore the relationships between exposure, religiosity, and distress between American Orthodox Jews	Cross-sectional study (*n* = 419 American Orthodox Jews)	DUREL Scale	Positive religious coping, intrinsic religiosity and trust in God emerged as strong correlates of less stress and increased positivity. The finding suggests that utilizing positive religious coping during the pandemic can provide mental health benefits	17.2/22 STROBE
Prazeres et al. (2021)	Describe the role of spiritual and religious coping in COVID-19-related fear and anxiety between healthcare professionals	Cross-sectional study (*n* = 222 healthcare professionals in Portugal)	Scales: DUREL, SS and CAS	Religiosity wasn’t a significant factor regarding to anxiety and fear of the coronavirus. However, spirituality was associated with lower coronavirus-related anxiety. Participants with higher levels of hope and optimism showed less coronavirus-related anxiety	18.7/22 STROBE
Prieto-Ursúa et al. (2020)	To analyze the presence of post-traumatic growth during the coronavirus crisis and to understand the contribution of meaning, religiosity, and spirituality to such growth	Cross-sectional study (*n* = 1091 Spanish citizens)	Scales: PIL and CPTG	There is a great distinction between Religiosity and Spirituality, each one has different roles in post-traumatic growth. In difficult times, as the one experienced, it’s more necessary for people to reflect on purposes and goals in life, the experience of transcendence and meaning, and social support, and thus increase resilience and the ability to overcome problems	18.4/22 STROBE
Rababa et al. (2021)	To examine the association of death anxiety with religious coping and spiritual well-being among older adults during the COVID-19 pandemic	Cross-sectional study (*n* = 248 elders from Jordan)	Scales: ASDA, SWBS and BARCS	Religious coping and spiritual well-being may be significant predictors of death anxiety in older adults, as people's spirituality increases, their fear levels decrease. Spiritual interventions can be effective for the elderly to be more functional, face their fears and experience the active aging process, so it’s advisable to identify the spiritual needs of the elderly and provide spiritual care	17.7/22 STROBE
Rajabipoor et al. (2021)	To identify the components of spirituality that affect the resilience of nurses in the coronavirus service	Qualitative study (*n* = 11 nurses)	Self-made surveys	Seven components which affects the resilience of nurses are religious values, ethical orientation, wisdom, voluntary activities, self-awareness, belief in the otherworld, patience, and hope	16/21 SRQR
Ren et al. (2021)	To explore the intervention degree and improvement effect of group reminiscence therapy in combination with physical exercise on spiritual well-being of the elderly after the outbreak of the COVID-19 epidemic	Randomized controlled trial (*n* = 130 elders, 65 in the experimental group and 65 in the control group)	Group reminiscence therapy + physical exercise, scales: SIWB, ULS and BRS	After the intervention, loneliness of the elderly decreased significantly. The sense of social connection among the elderly, led them to feel understood and respected. Besides, psychological resilience and spiritual well-being increased significantly. Therefore, physical exercise and reminiscence jointly promote mental health and spiritual well-being of the elderly	18/25 CONSORT
Rias et al. (2020)	to determine associations of knowledge, attitudes, and practices (KAP) and spirituality with anxiety among a population during the COVID-19 pandemic	Cross-sectional study (*n* = 1082 people from Indonesia)	Scales: DASS-21 and DSES	Spirituality, knowledge, attitudes, and practice are significantly related to decreased anxiety regarding COVID-19 in the general population. It is important to improve these factors as a therapeutic approach in order to reduce the anxiety levels of the population	18.9/22 STROBE
Roberto et al. (2020)	To investigate the association between spirituality, resilience, and coping for women during COVID-19	Mixed design (quali-quanti) (*n* = 88 women)	Scales: CD-RISC and DSES	The predominant finding was that participants’ faith and spirituality helped them in coping with the day-to-day experiences of living during a pandemic, as well as having hope for the future	17.5/22 STROBE
Saini et al. (2021)	To study the impact of subjective vitality on spiritual intelligence and estimating the impeding effect of stress on spiritual intelligence, subjective vitality, and mindfulness	Cross-sectional study (*n* = 473 workers)	Scales: SVS, FFMQ and SQ21	There is a positive impact of subjective vitality on spiritual intelligence and a significant negative effect of stress on spiritual intelligence, subjective vitality, and mindfulness. Using these stress relievers may help people introspect during the COVID-19 pandemic and better manage the psychological consequences of the crisis	16.7/22 STROBE
Schnell et al. (2020)	To document levels of acute COVID-19 stress and general mental distress during the lockdown and in the weeks thereafter	Cross-sectional study (*n* = 1527 German speaking people)	Scales: PHQ-4 and SCS-KD	Meaning and self-control may be buffers between COVID-19 stress and general mental distress: when COVID-19 stress is high, the presence of meaning and self-control helps to decrease mental distress levels. Additionally, people who were highly stressed by COVID-19 were more likely to develop a meaning crisis, which was associated with higher mental distress. Health professionals may advise people to face existential issues and struggles and encourage them to exercise self-control	18.5/22 STROBE
Thomas et al. (2020)	To explore positive religious coping among Muslims and Christians during the early stages of the national response to the COVID-19 pandemic	Cross-sectional study (*n* = 543 participants, 339 Muslims and 204 Christians)	Scales GAD7, PHQ8 and RCOPE-14	Positive religious coping during infectious disease outbreaks may help some individuals reduce their risk of depressive illness. Religious coping was inversely related to the current levels of depressive symptomatology and history of psychological disorder	18.2/22 STROBE

### Evaluation of Methodological Quality

Evaluation of the methodological quality of the selected investigations was carried out by using the tools contained in the Equator guidelines. Strobe (von Elm et al., 2014) for observational studies, Consort (Schulz et al., 2010) for clinical trials, and SRQR guidelines (O’Brien et al., 2014) for qualitative studies. (see supplementary material). This analysis was carried out by two authors (Author 2 and Author 3) and by a third author (Author 5) to resolve discrepancies.

## Results

**Fig. 1:**
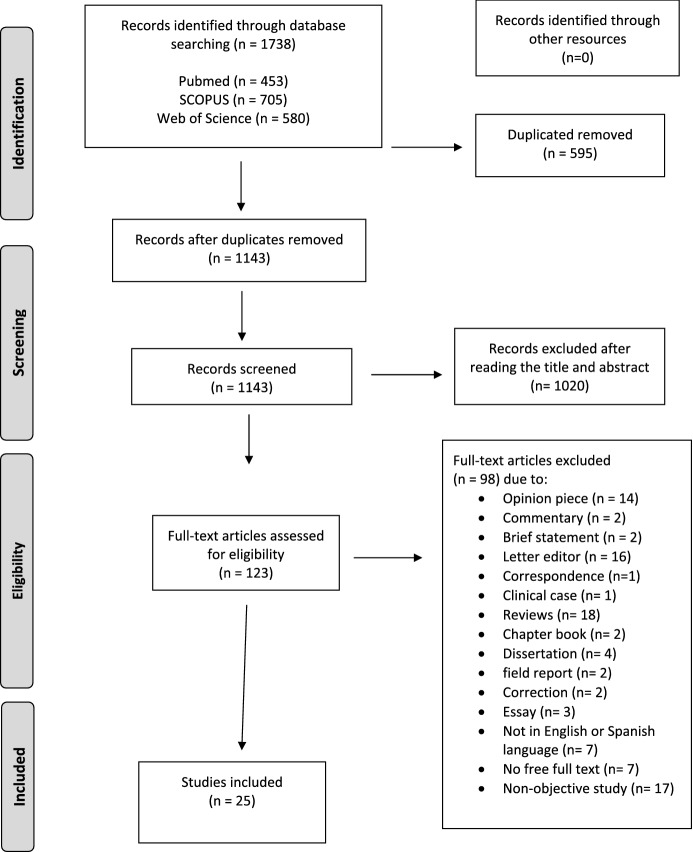
PRISMA flowchart

A flow chart was prepared according to the Prisma Declaration (Page et al., 2021). First, 1,338 articles were retrieved after applying the database search strategy. No results were extracted from the gray literature search. After removing duplicate records and reviewing the titles and abstracts of 1,143 records, a total of 123 studies were selected for full text reading (Fig. 1).

Finally, a total of 25 articles have been included in the review, of which 22 were descriptive observational studies, 2 were qualitative studies, and 1 was a randomized controlled study. A total of 88% of the studies included in this review adopted a descriptive cross-sectional design, 8% qualitative designs, and 4% clinical trials. The included studies are presented in Table 3.

### Quality and Characteristics Assessment of the Included Studies

The quality of the studies ranged from medium to good, with the observational studies obtaining scores on the Strobe statement more than 14 points out of 22, the qualitative studies obtained scores on the SRQR statement more than 16 points out of 21 and the clinical trial obtained results on the Consort statement more than 18 points out of 25.

The countries where the most studies were conducted were the USA (22%; *n* = 5), followed by Iran (12%; *n* = 3) and India (12%; *n* = 3). Regarding the population analyzed, 12% (*n* = 3) included older adults (Durmuş & Durar, 2021; Rababa et al., 2021; Ren et al., 2021). However, most articles (88%; *n* = 22) did not separate into specific population groups.

The sample size of the studies ranged from 18 (Hamilton et al., 2021) to 1,527 subjects (Schnell & Krampe, 2020). Most of the articles selected for this review investigated the effect of spirituality and spiritual care on people's health during the COVID-19 pandemic (56%; *n* = 14). However, others such as (Pirutinsky et al., 2021) focused their research on the religious needs of people during the COVID-19 pandemic (28%; *n* = 7), or analyzed both issues (16%; *n* = 4 studies). When dealing with religiosity, the predominant religion in the selected articles was Islam (Mahamid & Bdier, 2021; Mahmood et al., 2021; Rababa et al., 2021; Thomas & Barbato, 2020), followed by Christianity (Fatima et al., 2020; Kowalczyk et al., 2020; Lucchetti et al., 2020).

Regarding healthcare professionals, 16% of the articles studied the influence of spirituality on health professionals who worked during the COVID-19 pandemic, of which 75% included only nurses (Kim et al., 2021; Kostovich et al., 2021; Rajabipoor Meybodi & Mohammadi, 2021), and the remaining 25% did not specify the type of healthcare professionals they included (Prazeres et al., 2020).

### Spiritual Care Provided by Healthcare Professionals During the COVID-19 Pandemic

Our findings support the role of religious and spiritual coping for health professionals in overcoming challenges during stressful times, such as those suffered during the pandemic due to the high amount of workload and the high number of patients’ deaths. For example, Rajabipoor Meybodi and Mohammadi (2021) carried out a qualitative study, identifying that components of spirituality had an important influence on the resilience of nurses in a coronavirus service in Iran. They identified seven components that influenced nurses’ resilience: religious values, ethical orientation, wisdom, voluntary activities, self-awareness, belief in the otherworld, patience and hope. These components were intrinsically related to the spiritual views of the participants.

In the same line, Kim et al. (2021) compared nurses' mental health status before and during the COVID-19 pandemic using an online cross-sectional survey of 320 nurses from the USA. They found that greater levels of spirituality and a high family functioning were significant negative predictors of stress, anxiety and depression, while caring for COVID-19 patients and being in quarantine were significant positive predictors of stress and anxiety.

Prazeres et al. (2020) have also investigated the role of spiritual-religious coping in the fear and anxiety of COVID-19 among 222 healthcare workers (HCWs) in Portugal. It was observed that religiosity was neither a significant factor for coronavirus-related anxiety nor for fear of COVID-19. However, spirituality was associated with lower coronavirus-related anxiety. According to these authors, online religious and spiritual support for healthcare workers may be important strategies to promote spiritual-religious support during COVID-19 in this population.

Finally, a study has also assessed the role of spiritual/religious interventions on health outcomes in healthcare professionals. This study included 15 Registered nurses (RNs) working in acute care units of a Veteran Affairs Medical Center and 22 patients, who were recruited after the nurses had completed their Mantram Repetition Program (MRP) training. Two months after intervention, more than half of the RNs continued to use the MRP tools. There was a significant increase in peace as a dimension of spiritual well-being, in compassion satisfaction as a dimension of professional quality of life, and in mindfulness. The perceptions of the presence of nurses by the patients were very high and the patients also demonstrated high levels of satisfaction with the general nursing care (Kostovich et al., 2021).

### The Importance of Spiritual and Religious Beliefs Among the General Population During the Covid-19 Pandemic

#### Mental Health Problems

The COVID-19 pandemic resulted in significant mortality and morbidity worldwide. The devastating effect of the COVID-19 pandemic affected general well-being, including mental health (Fatima et al., 2020).

The association between religiosity and spirituality and the mental health consequences of social isolation during the COVID-19 pandemic in Brazil has been investigated. Their findings indicated that there was a high use of religious and spiritual beliefs during the pandemic and that this use was associated with better health outcomes, as evidenced by higher levels of hopefulness and lower levels of fear, worry and sadness in more religious and spiritual participants (Lucchetti et al., 2020).

Religious coping and spirituality were resources used to mitigate the effects the COVID-19 pandemic has caused on people’s mental health. Stress has been one of the most prevalent mental health problems during the COVID-19 pandemic and lockdown. The people of African-American breast cancer survivors used spirituality to cope with stressors during the pandemic and found several coping strategies such as increased participation in religious activities; reliance on God for protection when fearful, finding joy and courage from listening to gospel music and reading scripture; and finding meaning through spirituality (Hamilton et al., 2021).

Spiritual well-being and positive religious coping also have a great impact on anxiety and fear levels, in the sense that the higher the level of spiritual well-being an older adult has, the lower their level of death anxiety and fear (Durmuş & Durar, 2021; Mahmood et al., 2021; Rababa et al., 2021). According to (Rababa et al., 2021), when older adults have good spiritual health, they experience positive feelings about their current situation and a strong inner healing force, identified as a coping strategy to deal with the actual situation. The results found by (Rias et al., 2020) affirm that people who had knowledge, confidence in 'winning' the battle against disease and higher spirituality had lower levels of anxiety.

Our analysis showed that various studies (Mahamid & Bdier, 2021; Pirutinsky et al., 2021; Thomas & Barbato, 2020) have similar results. Positive religious/spiritual coping decreased levels of stress, anxiety, and depressive symptoms and increased positive emotions, providing health benefits during the pandemic. For example, a previous study found that spiritual health was associated with greater levels of self-efficacy among pregnant women which, in turn, was associated with better mental health and lower probability of suffering from preterm and unhealthy labor (Nodoushan et al., 2020).

Finally, other studies found that a high degree of subjective vitality, mindfulness, and spiritual intelligence (Saini & Seema, 2021), and the presence of meaningfulness and self-control (Schnell & Krampe, 2020) may be considered great resources to decrease levels of mental distress. However, (Nooripour et al., 2021) found that spiritual well-being itself did not predict stress from COVID-19 alone, but along with hope and resilience, which are also good predictors of stress.

#### Well-Being

Some studies included in the research investigated the effect of religiosity and spirituality on well-being. According to Fatima et al. (2020), people use religious and spiritual coping strategies to stop worrying about their problems, to ask for forgiveness, to handle stressful situations and to manage anger during the COVID-19 pandemic. People experiencing fear, suffering, or illness often experience a 'spiritual renewal', because faith allows people to keep hope and feel a sense of security (Kowalczyk et al., 2020).

In the same line, (Al Eid et al., 2021) showed that religiosity had a direct positive effect on the psychological well-being of COVID-19 patients, suggesting that the greater the individual’s religiosity, the greater his psychological well-being.

The role of spirituality and religiosity in well-being was also observed in other studies. A study identified that participants’ faith and spirituality helped them to cope with the day-to-day experiences of living during a pandemic (Roberto et al., 2020). Another study included 292 oncology patients and found that they perceived some changes in their attitudes during the COVID-19 pandemic that also contributed to their well-being: the importance of meaning in life, having (religious) trust, stable relationships, mindful encounter with nature, and having times of reflection (Büssing et al., 2020).

Spirituality and religiosity may also affect the post-traumatic growth of people during the COVID-19 pandemic. A study wanted to identify the role of spirituality and religiosity in posttraumatic growth, finding that each has different roles in posttraumatic growth. Perceived spirituality broadly coincides with meaning in predicting growth. Perceived religiosity, on the other hand, seems to contribute other significant values and models in addition to meaning, which facilitate social and interpersonal growth in the face of traumatic and life-threatening situations. The results confirm the importance of meaning in posttraumatic growth, especially the dimension of life goals and purposes. Even in situations as difficult as the one experienced, with the immediate threat of death and disease, during a strict lockdown, surrounded by pain and fear, it is possible, and more necessary than ever, that people reflect on purposes and goals in life, the experience of transcendence and meaning, and social support (Prieto-Ursúa & Jódar, 2020).

Finally, there are some intervention studies that support the observational findings. A study investigated the impact of transcendent yoga practice in a group of 126 people in India, finding that after 30 days of practicing yoga exercises, most of the participants reported that their stress level decreased, they felt more energetic, their flexibility increased and they had better sleep habits (Malik & Sharma, 2020). In another study, conducted a randomized controlled trial with the aim of exploring the degree of intervention and the effect of group reminiscence therapy in combination with physical exercise on spiritual well-being of older adults after the outbreak of the COVID-19 epidemic. According to the study results, after the intervention, loneliness decreased significantly, and the sense of social connection led them to feel understood and respected. In addition, psychological resilience and spiritual well-being increased significantly (Ren et al., 2021). Therefore, physical exercise and reminiscence together may promote the spiritual well-being during the pandemic.

## Discussion

The purpose of this review was to analyze the role of spirituality and religiosity on health care during the COVID-19 pandemic. Most studies investigated the effect of spiritual/religious beliefs on people's health during the COVID-19 pandemic. Other studies have focused on spiritual interventions and on the use of coping strategies by healthcare professionals. Our findings denote the importance of addressing spiritual needs in clinical practice due to its benefits, as well as highlight the need for training health professionals to be able to carry out interventions that take into account the spiritual and religious aspects.

First, in studies that evaluate healthcare professionals, our findings revealed that spiritual and religious issues are important aspects for these professionals and can influence their health and clinical practice. Some articles have emphasized that the mental health of professionals has been affected by the pandemic and identified spirituality and good family functioning (Kim et al., 2021), as well as religious or spiritual beliefs and practices (Sierra Leguía & Montalvo Prieto, 2012) and faith (Matheson et al., 2020) as coping strategies. This is fully supported by previous studies before the pandemic, which have also found that religiosity and spirituality were significantly associated with a reduction in anxiety levels and an improvement in depressive symptoms (Gonçalves et al., 2015; Hook et al., 2010; Peselow et al., 2014).

In relation to the resilience of nurses during the pandemic, religious values, morality, self-awareness, patience and hope, wisdom, voluntary activities and belief in the afterlife have been identified as important components of being resilient (Rajabipoor Meybodi & Mohammadi, 2021; Wei et al., 2019). Together with resilience, spiritual practices appeared to provide a sense of security and inner peace that prevent the appearance of other negative emotions such as fear, anxiety, or insecurity (Prazeres et al., 2020), although some studies also suggest that nurses with greater spiritual perception present high levels of anxiety before death (Rahman et al., 2021).

It is interesting to note that some interventions designed to promote spiritual and religious beliefs (e.g. mantra repetition programs (Kostovich et al., 2021)) appeared to be effective for nurses during the pandemic, as this was in line with the interventions offered before the pandemic for those professionals such as the creation of a good spiritual work environment (Wu et al., 2020), prayer (Ibrahim et al., 2020) or spirituality training programs on well-being and spiritual integrity (Yong et al., 2011). In this regard, previous studies have pointed to the need to include religiosity and spirituality education programs for undergraduate and graduate students, providing relevant training courses for nurses that allow them to learn the skills necessary to provide spiritual care and to handle their spiritual needs (de Diego Cordero et al., 2019; Moreira-Almeida et al., 2014).

Although the literature highlights the unpreparedness and burnout of health professionals while addressing spiritual needs in the hospitals due to the risks of contagion of chaplains (Chirico & Nucera, 2020), it is important to highlight that chaplains were and are very important figures in the COVID-19 pandemic as well. According to the different communities and contexts, chaplains have responded in different ways, providing emotional, religious, spiritual support during this challenging moment where gathering have been prohibited in many places (Carey et al., 2020). These risks have lead chaplains to provide spiritual care at a distance, trying to maintain contact with the believers, shifting towards online-based services, spiritual recollections and retreats, community prayers and sacraments (Domaradzki, 2022). Likewise, religious leaders were allowed to use online devices to provide spiritual care during the hospitalization to alleviate the suffering of patients. These strategies were essential to minimize the problems arising from the uncertainty of the disease (Badanta et al., 2021).

Second, several studies have assessed the role of spirituality and religiosity in different health outcomes for the general population during the COVID 19 pandemic. These studies have revealed that spiritual and religious beliefs could be associated with greater coping, less mental health problems (stress, anxiety, depression) and better well-being. According to previous studies, the most prevalent negative emotional symptoms during the pandemic were anguish, fear, and suffering. In this context, spirituality appears to be an important tool for overcoming suffering among individuals. These results found during the pandemic were also observed before the pandemic by several studies. Individuals tend to use religiosity and spirituality to face problems, with actions such as greater participation in religious activities, trust in God for their protection against fear and isolation; find joy and courage when listening to gospel music and reading scriptures (Hamilton et al., 2021), using positive religious coping strategies (Fatima et al., 2020; Mahamid & Bdier, 2021; Thomas & Barbato, 2020), trusting in God (Pirutinsky et al., 2021), supporting their closest loved ones, using spiritual intelligence, subjective vitality and mindfulness (Saini & Seema, 2021). All these strategies were associated with decreased fear, relief from suffering and increased well-being (Durmuş & Durar, 2021; Lucchetti et al., 2020; Rababa et al., 2021), experiencing 'spiritual renewal' (Kowalczyk et al., 2020). These results could serve to make healthcare professionals and healthcare managers aware of the spiritual and religious needs of their patients, to promote integrative and person-centered care. They should ensure the continuity of religiosity and spirituality activities during the pandemic (Lucchetti et al., 2020) and consider these factors when planning interventions to address health problems in times of crisis in the treatment of mental health (Mahmood et al., 2021; Moreira-Almeida et al., 2014).

### Study Limitations

Our review has some limitations that should be mentioned. First, the number of publications on COVID-19 is rapidly evolving and, for this reason, it is probable that some studies have been published after our search and were not included. Second, we have included three databases. Therefore, some articles indexed in other databases were not included. Finally, our review did not include letters to the editors, commentaries, and theses. In particular, at the beginning of the pandemic, several letters were published concerning spiritual issues with some preliminary results. However, these letters were not included because there was no way to evaluate the quality of the methods used in these studies.

## Conclusions

In conclusion, our findings revealed that spirituality could be considered a good coping strategy used by healthcare professionals to deal with mental health problems during the COVID-19 pandemic, providing greater inner strength, resilience and well-being, as well as greater patient satisfaction with the care given. In relation to the general population, evidence shows that meeting the spiritual needs of individuals leads to a reduction in stress, anxiety, depression, and other negative emotional symptoms that have appeared in people during the COVID-19 pandemic, thus achieving an increase in psychological well-being and providing resilience and hope.

For both healthcare professionals and the general population, spirituality has helped reduce negative emotional symptoms related to the COVID-19 pandemic, providing psychological well-being, resilience, and hope. Our results support the need for adequate spirituality training for health professionals, aiming to promote integrative care that takes into account the spiritual and religious aspects of people.

## Supplementary Information

Below is the link to the electronic supplementary material.

Supplementary file1 (DOCX 36 KB)

Supplementary file2 (DOCX 21 KB)

Supplementary file3 (DOCX 17 KB)
