# Supplementary material for: The Role of Spirituality and Religiosity in Healthcare During the COVID-19 Pandemic: An Integrative Review of the Scientific Literature
Source: J Relig Health. 2022 Mar 29;61(3):2168–97. doi: 10.1007/s10943-022-01549-x (PMC8960681; doi:10.1007/s10943-022-01549-x)
Supplement: Supplementary file 1 — Supplementary file1 (DOCX 36 KB) [file 10943_2022_1549_MOESM1_ESM.docx]

**Table S1.** Quality evaluation of the studies included in the systematic review (STROBE).

|  | **Title/abstract** | | **Background /** **rationale** | **Objectives** | **Study design** | **Setting** | **Participants** | **Variables** | **Data sources /** **measurement** | **Bias** | **Study size** | **Quantitative variables** | **Statistical**  **methods** | | | | |
| --- | --- | --- | --- | --- | --- | --- | --- | --- | --- | --- | --- | --- | --- | --- | --- | --- | --- |
|  | 1 | | 2 | 3 | 4 | 5 | 6 | 7 | 8 | 9 | 10 | 11 | 12 | | | | |
|  | a | b |  |  |  |  |  |  |  |  |  |  | a | b | c | d | e |
| Al Eid et al.^39^ | 0 | 0.5 | 1 | 1 | 1 | 0.5 | 1 | 1 | 1 | 0 | 0.5 | 1 | 0.2 | 0.2 | 0 | 0.2 | 0 |
| Büssing et al.^41^ | 0 | 0.5 | 1 | 1 | 1 | 1 | 1 | 1 | 1 | 0 | 1 | 1 | 0.2 | 0.2 | 0 | 0.2 | 0 |
| Durmus et al.^21^ | 0.5 | 0.5 | 1 | 1 | 1 | 1 | 1 | 1 | 1 | 0 | 1 | 1 | 0.2 | 0 | 0 | 0.2 | 0 |
| Fatima et al.^30^ | 0.5 | 0.5 | 1 | 1 | 1 | 1 | 1 | 1 | 1 | 0 | 1 | 1 | 0.2 | 0.2 | 0 | 0.2 | 0 |
| Kim et al.^32^ | 0.5 | 0.5 | 1 | 1 | 1 | 1 | 1 | 1 | 1 | 0 | 1 | 1 | 0.2 | 0.2 | 0 | 0 | 0.2 |
| Kostovich et al.^31^ | 0.5 | 0.5 | 1 | 1 | 1 | 1 | 1 | 1 | 1 | 1 | 1 | 1 | 0.2 | 0.2 | 0.2 | 0.2 | 0 |
| Kowalczyk et al.^29^ | 0 | 0 | 1 | 1 | 1 | 1 | 1 | 1 | 1 | 0 | 1 | 1 | 0.2 | 0 | 0 | 0.2 | 0 |
| Lucchetti et al.^28^ | 0.5 | 0.5 | 1 | 1 | 1 | 1 | 1 | 1 | 1 | 0 | 1 | 1 | 0.2 | 0.2 | 0.2 | 0.2 | 0 |
| Mahamid et al.^27^ | 0.5 | 0.5 | 1 | 1 | 1 | 1 | 1 | 1 | 1 | 0 | 1 | 1 | 0.2 | 0 | 0 | 0.2 | 0 |
| Mahmood et al.^26^ | 0.5 | 0.5 | 1 | 1 | 1 | 1 | 1 | 1 | 1 | 0 | 1 | 1 | 0.2 | 0.2 | 0 | 0.2 | 0 |
| Malik et al.^43^ | 0 | 0 | 1 | 1 | 1 | 0 | 1 | 1 | 1 | 0 | 1 | 1 | 0.2 | 0 | 0 | 0.2 | 0 |
| Nodoushan et al.^36^ | 0 | 0.5 | 1 | 1 | 1 | 1 | 1 | 1 | 1 | 0 | 1 | 1 | 0.2 | 0 | 0 | 0.2 | 0 |
| Nooripour et al.^38^ | 0 | 0.5 | 1 | 1 | 1 | 1 | 1 | 1 | 1 | 0 | 1 | 1 | 0.2 | 0.2 | 0 | 0.2 | 0 |
| Pirutinsky et al.^24^ | 0 | 0.5 | 1 | 1 | 1 | 1 | 1 | 1 | 1 | 0 | 1 | 1 | 0.2 | 0.2 | 0 | 0.2 | 0 |
| Prazeres et al.^34^ | 0.5 | 0.5 | 1 | 1 | 1 | 1 | 1 | 1 | 1 | 0 | 1 | 1 | 0.2 | 0.2 | 0 | 0.2 | 0 |
| Prieto-Ursúa et al.^42^ | 0 | 0.5 | 1 | 1 | 1 | 1 | 1 | 1 | 1 | 0 | 1 | 1 | 0.2 | 0.2 | 0.2 | 0.2 | 0 |
| Rababa et al.^20^ | 0.5 | 0.5 | 1 | 1 | 1 | 1 | 1 | 1 | 1 | 0 | 1 | 1 | 0.2 | 0.2 | 0 | 0.2 | 0 |
| Rias et al.^35^ | 0.5 | 0.5 | 1 | 1 | 1 | 1 | 1 | 1 | 1 | 0 | 1 | 1 | 0,2 | 0.2 | 0.2 | 0.2 | 0 |
| Roberto et al.^40^ | 0.5 | 0.5 | 1 | 1 | 1 | 1 | 1 | 1 | 1 | 0 | 1 | 1 | 0.2 | 0 | 0 | 0.2 | 0 |
| Saini et al.^37^ | 0 | 0.5 | 1 | 1 | 1 | 1 | 1 | 0 | 1 | 0 | 1 | 1 | 0.2 | 0.2 | 0.2 | 0.2 | 0 |
| Schnell et al.^23^ | 0.5 | 0.5 | 1 | 1 | 1 | 1 | 1 | 1 | 1 | 0 | 1 | 1 | 0.2 | 0.2 | 0 | 0.2 | 0 |
| Thomas et al.^25^ | 0 | 0.5 | 1 | 1 | 1 | 1 | 1 | 1 | 1 | 0 | 1 | 1 | 0.2 | 0.2 | 0 | 0.2 | 0 |

(continuation)

|  | **Participants (results)** | | | **Descriptive** **data** | | **Outcome data** | **Main results** | | | **Other analyses** | **Key results** | **Limitations** | **Interpretation** | **Generalisability** | **Funding** | **Sum** |
| --- | --- | --- | --- | --- | --- | --- | --- | --- | --- | --- | --- | --- | --- | --- | --- | --- |
|  | 13 | | | 14 | | 15 | 16 | | | 17 | 18 | 19 | 20 | 21 | 22 |  |
|  | a | b | c | a | b |  | a | b | c |  |  |  |  |  |  |  |
| Al Eid et al.^39^ | 0.3 | 0 | 0.3 | 0.5 | 0 | 1 | 0.3 | 0.3 | 0 | 1 | 1 | 1 | 1 | 1 | 1 | **17.8** |
| Büssing et al.^41^ | 0.3 | 0.3 | 0 | 0.5 | 0 | 1 | 0.3 | 0.3 | 0 | 1 | 1 | 1 | 1 | 1 | 0 | **17.8** |
| Durmus et al.^21^ | 0.3 | 0 | 0 | 0.5 | 0 | 1 | 0.3 | 0.3 | 0 | 0 | 1 | 1 | 1 | 1 | 1 | **17.8** |
| Fatima et al.^30^ | 0.3 | 0.3 | 0 | 0.5 | 0 | 1 | 0.3 | 0.3 | 0 | 1 | 1 | 1 | 1 | 1 | 1 | **19.3** |
| Kim et al.^32^ | 0.3 | 0 | 0 | 0.5 | 0.5 | 1 | 0.3 | 0.3 | 0 | 1 | 1 | 1 | 1 | 1 | 0 | **18.5** |
| Kostovich et al.^31^ | 0.3 | 0.3 | 0 | 0.5 | 0.5 | 1 | 0.3 | 0.3 | 0 | 1 | 1 | 1 | 1 | 1 | 1 | **21** |
| Kowalczyk et al.^29^ | 0.3 | 0.3 | 0 | 0.5 | 0.5 | 1 | 0.3 | 0 | 0 | 0 | 1 | 0 | 1 | 1 | 0 | **15.3** |
| Lucchetti et al.^28^ | 0.3 | 0.3 | 0 | 0.5 | 0.5 | 1 | 0.3 | 0 | 0 | 1 | 1 | 1 | 1 | 1 | 1 | **19.7** |
| Mahamid et al.^27^ | 0.3 | 0 | 0 | 0.5 | 0 | 1 | 0.3 | 0 | 0 | 1 | 1 | 1 | 1 | 1 | 0 | **17.5** |
| Mahmood et al.^26^ | 0.3 | 0 | 0.3 | 0.5 | 0 | 1 | 0.3 | 0 | 0 | 1 | 1 | 1 | 1 | 1 | 1 | **19** |
| Malik etal.^43^ | 0.3 | 0 | 0 | 0.5 | 0 | 1 | 0.3 | 0 | 0 | 0 | 1 | 1 | 1 | 1 | 0 | **14.5** |
| Nodoushan et al.^36^ | 0.3 | 0.3 | 0 | 0.5 | 0 | 1 | 0.3 | 0 | 0 | 1 | 1 | 1 | 1 | 0 | 0 | **16.3** |
| Nooripour et al.^38^ | 0.3 | 0 | 0 | 0,5 | 0 | 1 | 0.3 | 0 | 0 | 1 | 1 | 1 | 1 | 1 | 1 | **18.2** |
| Pirutinsky et al.^24^ | 0.3 | 0 | 0 | 0.5 | 0 | 1 | 0.3 | 0 | 0 | 1 | 1 | 1 | 1 | 1 | 0 | **17.2** |
| Prazeres et al.^34^ | 0.3 | 0 | 0 | 0.5 | 0 | 1 | 0.3 | 0 | 0 | 1 | 1 | 1 | 1 | 1 | 1 | **18.7** |
| Prieto-Ursúa et al.^42^ | 0.3 | 0 | 0 | 0.5 | 0 | 1 | 0.3 | 0 | 0 | 1 | 1 | 1 | 1 | 1 | 1 | **18.4** |
| Rababa et al.^20^ | 0.3 | 0 | 0 | 0.5 | 0 | 1 | 0.3 | 0 | 0 | 1 | 1 | 1 | 1 | 0 | 1 | **17.7** |
| Rias et al.^35^ | 0.3 | 0 | 0 | 0.5 | 0 | 1 | 0.3 | 0 | 0 | 1 | 1 | 1 | 1 | 1 | 1 | **18.9** |
| Roberto et al.^40^ | 0.3 | 0 | 0 | 0.5 | 0 | 1 | 0.3 | 0 | 0 | 1 | 1 | 1 | 1 | 1 | 0 | **17.5** |
| Saini et al.^37^ | 0.3 | 0 | 0.3 | 0.5 | 0 | 1 | 0.3 | 0 | 0 | 1 | 1 | 1 | 1 | 1 | 0 | **16.7** |
| Schnell et al.^23^ | 0.3 | 0.3 | 0 | 0.5 | 0.5 | 1 | 0.3 | 0 | 0 | 1 | 1 | 1 | 1 | 1 | 0 | **18.5** |
| Thomas et al.^25^ | 0.3 | 0 | 0 | 0.5 | 0 | 1 | 0.3 | 0 | 0 | 1 | 1 | 1 | 1 | 1 | 1 | **18.2** |

1= recommendation contained in the study, 0= recommendation not included, NA= not applicable.
