# Supplementary material for: The Role of Spirituality and Religiosity in Healthcare During the COVID-19 Pandemic: An Integrative Review of the Scientific Literature
Source: J Relig Health. 2022 Mar 29;61(3):2168–97. doi: 10.1007/s10943-022-01549-x (PMC8960681; doi:10.1007/s10943-022-01549-x)
Supplement: Supplementary file 2 — Supplementary file2 (DOCX 21 KB) [file 10943_2022_1549_MOESM2_ESM.docx]

**Table S2**. Quality evaluation of the studies included in the systematic review (CONSORT)

|  | **Title** | **Abstract** | **Background** | **Objectives** | **Trial design** | **Important changes to methods** | **Participants** | **Locations where data were collected** | **Interventions** | **Outcomes** | **Changes to trial outcomes** | **Sample size** | **Interim analyses and stopping guidelines** | **Randomisation: sequence generation** | **Type of randomisation; details of any restriction** | **Allocation**  **concealment mechanism** | **Implementation** | **Blinding** | **Description of the similarity of interventions** |
| --- | --- | --- | --- | --- | --- | --- | --- | --- | --- | --- | --- | --- | --- | --- | --- | --- | --- | --- | --- |
|  | 1 | | 2 | | 3 | | 4 | | 5 | 6 | | 7 | | 8 | | 9 | 10 | 11 | |
|  | a | b | a | b | a | b | a | b |  | a | b | a | b | a | b |  |  | a | b |
| Ren et al.^19^ | 0 | 0.5 | 0.5 | 0.5 | 0.5 | 0.5 | 0.5 | 0.5 | 1 | 0.5 | 0 | 0.5 | 0.5 | 0.5 | 0.5 | 1 | 1 | 0.5 | 0 |

(continuation)

| **Statistical methods** | **Methods for additional analyses** | **Participant flow (diagram)** | **Losses and exclusions after randomisation** | **Recruitment** | **Why the trial ended or was stopped** | **Baseline data** | **Numbers analysed** | **Outcomes and estimation** | **Binary outcomes** | **Ancillary analyses** | **Harms** | **Limitations** | **Generalisability** | **Interpretation** | **Registration** | **Protocol** | **Funding** | **Sum** |
| --- | --- | --- | --- | --- | --- | --- | --- | --- | --- | --- | --- | --- | --- | --- | --- | --- | --- | --- |
| 12 | | 13 | | 14 | | 15 | 16 | 17 | | 18 | 19 | 20 | 21 | 22 | 23 | 24 | 25 |  |
| a | b | a | b | a | b |  |  | a | b |  |  |  |  |  |  |  |  |  |
| 0.5 | 0 | 0.5 | 0.5 | 0.5 | 0 | 1 | 1 | 0.5 | 0 | 1 | 0 | 1 | 0 | 1 | 0 | 1 | 0 | **18** |

1= recommendation contained in the study, 0= recommendation not included, NA= not applicable.
