# Supplementary material for: The Role of Spirituality and Religiosity in Healthcare During the COVID-19 Pandemic: An Integrative Review of the Scientific Literature
Source: J Relig Health. 2022 Mar 29;61(3):2168–97. doi: 10.1007/s10943-022-01549-x (PMC8960681; doi:10.1007/s10943-022-01549-x)
Supplement: Supplementary file 3 — Supplementary file3 (DOCX 17 KB) [file 10943_2022_1549_MOESM3_ESM.docx]

**Table S3**. Quality evaluation of the studies included in the systematic review (SRQR).

|  | **Title** | **Abstract** | **Problem formulation** | **Purpose or research question** | **Qualitative approach and research paradigm** | **Researcher characteristics and reflexivity** | **Context** | **Sampling strategy** | **Ethical issues pertaining to human subjects** | **Data collection methods** | **Data collection instruments and technologies** |
| --- | --- | --- | --- | --- | --- | --- | --- | --- | --- | --- | --- |
|  | 1 | 2 | 3 | 4 | 5 | 6 | 7 | 8 | 9 | 10 | 11 |
| Hamilton et al.^22^ | 1 | 1 | 1 | 1 | 0 | 1 | 1 | 1 | 0 | 1 | 1 |
| Rajabipoor et al.^33^ | 0 | 1 | 1 | 1 | 0 | 0 | 1 | 1 | 1 | 1 | 1 |
| Roberto et al.^40^ | 1 | 1 | 1 | 1 | 1 | 0 | 1 | 1 | 0 | 1 | 1 |

(continuation)

| **Units of study** | **Data processing** | **Data analysis** | **Techniques to enhance trustworthiness** | **Synthesis and interpretation** | **Links to empirical data** | **Integration with prior work** | **Limitations** | **Conflicts of interest** | **Funding** | **Sum** |
| --- | --- | --- | --- | --- | --- | --- | --- | --- | --- | --- |
| 12 | 13 | 14 | 15 | 16 | 17 | 18 | 19 | 20 | 21 |  |
| 1 | 1 | 1 | 1 | 1 | 1 | 1 | 1 | 1 | 0 | **18** |
| 1 | 1 | 1 | 1 | 1 | 0 | 1 | 1 | 1 | 0 | **16** |
| 1 | 1 | 1 | 1 | 1 | 1 | 1 | 1 | 0 | 1 | **18** |

1= recommendation contained in the study, 0= recommendation not included, NA= not applicable.
